# Miller Fisher syndrome, Bickerstaff brainstem encephalitis and Guillain-Barré syndrome overlap with persistent non-demyelinating conduction blocks: a case report

**DOI:** 10.1186/s12883-018-1104-6

**Published:** 2018-07-21

**Authors:** Angela Puma, Jeanne Benoit, Sabrina Sacconi, Antonino Uncini

**Affiliations:** 1 Peripheral Nervous System, Muscle & ALS Department, University of Nice and Côte d’Azur (UCA), Nice, France; 2 UMR7370 CNRS, LP2M, Faculté de Médecine, University Côte d’Azur, Nice, France; 3 Université Côte d’Azur, Centre National de la Recherche Scientifique, Institut de Pharmacologie Moléculaire et Cellulaire, Valbonne, France; 4 Department of Neuroscience, Imaging and Clinical Sciences, University “G. d’Annunzio”, Chieti-Pescara, Italy

**Keywords:** Guillain-Barré syndrome, Miller Fisher syndrome, Bickerstaff brainstem encephalitis, Anti-GQ1b antibody, Nodopathy, Persistent motor conduction block

## Abstract

**Background:**

Miller Fisher syndrome (MFS) and Bickerstaff’s Brainstem Encephalitis (BBE) share some clinical features and a common immunological profile characterized by anti-GQ1b antibodies. Some MFS patients overlap with Guillain-Barré syndrome (GBS) or BBE. We report a patient with MFS, BBE, and axonal GBS overlap in whom serial electrophysiological studies showed persistent motor conduction blocks (CBs).

**Case presentation:**

A 61-year-old man acutely developed ophtalmoparesis, ataxia and areflexia suggesting MFS. Paresthesias, severe weakness, and drowsiness rapidly developed indicating an overlap with BBE and GBS. Preceding infection with *Mycoplasma Pneumoniae* and anti-GQ1b antibodies were detected. On day 4, nerve conduction study showed reduced or non-recordable compound muscle action potentials (CMAPs) and sensory nerve action potentials (SNAPs) without demyelinating features, indicating the electrodiagnosis of acute motor and sensory axonal neuropathy and suggesting a poor prognosis. Intravenous immunoglobulins (IVIg) were given but clinical status worsened to ophthalmoplegia, tetraplegia and coma needing mechanical ventilation. A second IVIg course was given and the patient was weaned off ventilation on day 41 and transferred to rehabilitation on day 57 with partial resolution of the ophthalmoplegia and limited recovery of muscle strength. Electrophysiology showed, after 10 weeks, greatly improved distal CMAP amplitudes suggesting the resolution of distal CBs while CBs in intermediate and proximal nerve segments emerged. CBs unusually persisted for four to 6 months without development of abnormal temporal dispersion. A third IVIg course was started on day 179 and the resolution of CBs mirrored the clinical improvement.

**Conclusions:**

GQ1b gangliosides are expressed in the nodal region of oculomotor nerves, muscle spindle afferents, peripheral nerves and possibly in the brainstem reticular formation. Anti-GQ1b antibodies may explain the complex symptomatology and the overlap between MFS, BBE, and GBS.

CBs that persisted and recovered without the development of temporal dispersion suggest that weakness was due to a sustained, antibody-mediated, attack at the nodal region inducing a non-demyelinating conduction failure as expression of an acute onset, long lasting, nodopathy. Serial electrophysiological studies allowed not only to understand the underlying pathophysiology and formulate a more correct prognosis but also to guide the treatment.

## Background

In the 1950s, Miller Fisher Syndrome (MFS) and Bickerstaff brain encephalitis (BBE) were independently described [1, 2]. Both disorders were related a preceding infection, showed ophthalmoplegia and ataxia, and presented a spontaneous recovery in most cases. MFS patients were areflexic, in keeping with a peripheral nerve etiology, whereas BBE was characterized by altered consciousness and hyperreflexia in some patients, supporting a central pathology. Interestingly, both Fisher and Bickerstaff recognized in the diseases they described similarities with Guillain–Barré syndrome (GBS) [1, 2].

In the early 1990s, the discovery of anti-GQ1b IgG antibodies in MFS and BBE provided evidence that both disorders were part of the same disease spectrum, later referred to as anti-GQ1b antibody syndrome [3–5]. MFS patients who develop limb weakness overlapping with Guillain Barré syndrome (GBS), or drowsiness overlapping with BBE [6, 7] also support a common immunological profile and clinical spectrum.

We report a patient who developed, after a *Mycoplasma Pneumoniae* infection, an overlap of MFS, BBE and axonal GBS with GQ1b IgG antibodies and showing, at serial conduction studies, persistent motor conduction blocks (CB).

## Case presentation

A 61-year-old man acutely developed diplopia and balance disorders. 24 h after onset, he was admitted in the hospital due to ascending paresthesias and an emerging tetraparesis. On day 4 after onset, examination showed a drowsy patient with ophthalmoparesis, areflexia and severe tetraparesis. Plantar responses were flexor. The cerebrospinal fluid study was normal. IgM to *Mycoplasma Pneumoniae* and IgG anti-GQ1b (1:2560) were detected. IgG and IgM anti- GM1, −GD1a, −GD1b and -GM2 were absent. Two MRIs of the brain, one with gadolinium, were normal. The patient was treated with intravenous immunoglobulin (IVIg) (0,4 g/kg/day X 5 days) but worsened to complete ophthalmoplegia, tetraplegia and coma needing mechanical ventilation. Considering the severity of the condition a second IVIg course was started on day 24. A few days later he began to improve, with initial resolution of the drowsiness and bulbar symptoms, and partial resolution of the ophtalmoplegia. The patient was weaned off ventilation on day 41 and transferred to rehabilitation on day 57 with improved muscle strength (MCR scale: 2–3/5 in the lower and 2/5 in the upper limbs). After considering the results of the nerve conduction studies performed on days 72 and 128, we searched for antibodies to Neurofascin155, Contactin1 and Contactin associated protein1 that resulted negative. On the other hand, five months after the onset of symptoms, the anti GQ1b IgG rate remained elevated (1:2560).

A third IVIg course was started on day 179. On day 240, the patient had recovered muscle strength, except in the right upper limb (MCR scale: 3–4/5) and showed mild gait ataxia.

### Nerve conduction studies

**Fig. 1:**
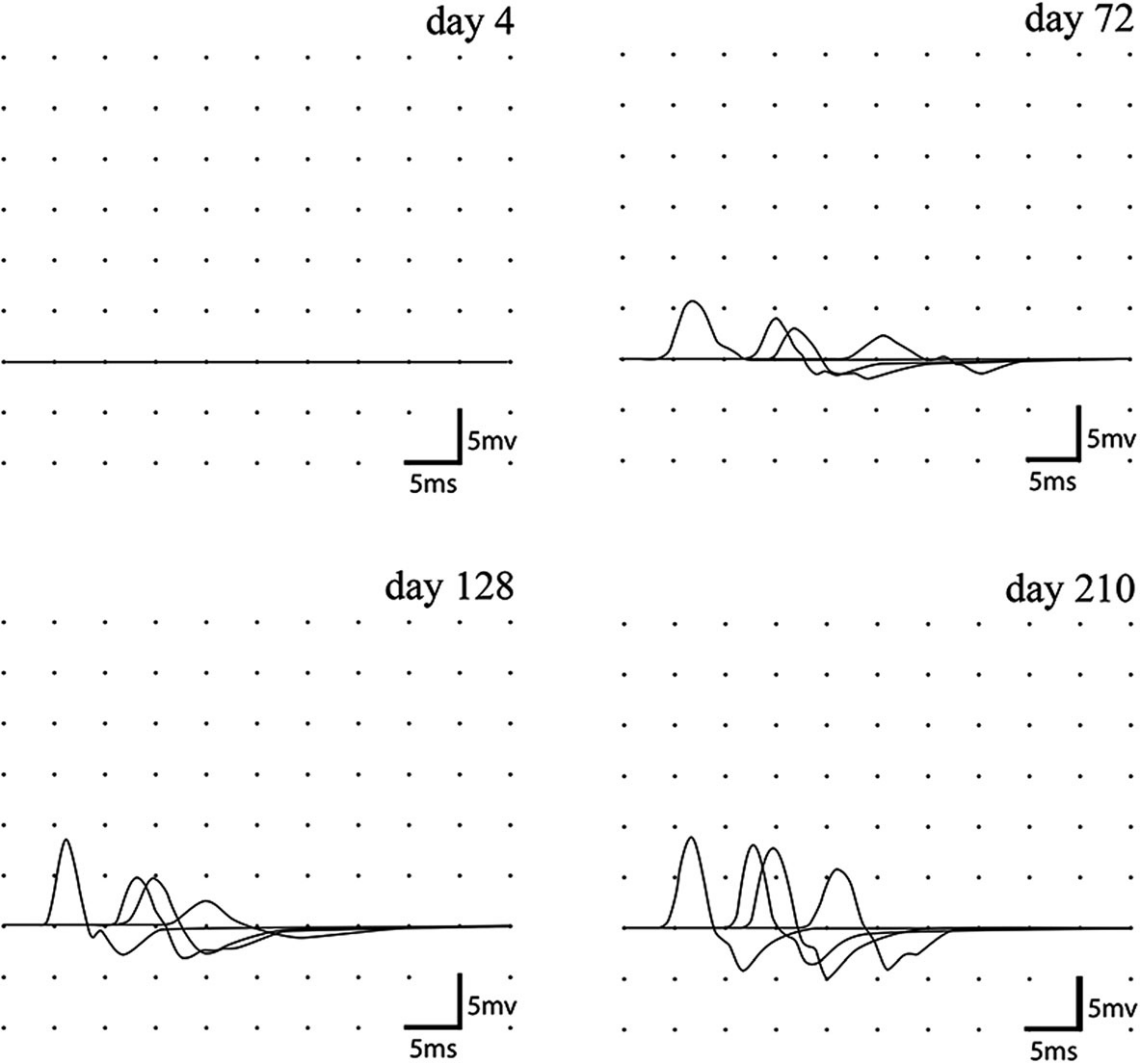
Serial motor conduction recordings of the right median nerve. Superimposed compound muscle action potentials (CMAPs) recorded from the abductor pollicis brevis after median nerve stimulation at wrist, elbow, axilla and Erb’s point. On day 4, distal CMAP was not recordable. On day 72, distal CMAP was 5,3 mV and partial CB was detectable in the elbow-wrist segment (proximal CMAP amplitude reduction = 30,2%; proximal CMAP area reduction = 51,3%) and in the Erb’s point-axilla segment (proximal CMAP amplitude reduction = 35%; proximal CMAP area reduction = 30%). On day 128 distal CMAP amplitude was 8.3 mV but CBs persisted. The last recording on day 210 showed resolution of CBs. In all recordings there was no evidence of CMAP abnormal temporal dispersion

Four serial conduction studies were performed (Table 1, Fig. 1). On day 4 compound muscle action potentials (CMAPs) were not recordable or were very reduced in amplitudes with slightly prolonged distal motor latencies (DML) and normal motor conduction velocities (MCVs). Sensory nerve action potentials (SNAPs) showed reduced amplitudes in the right sural nerve and were not recordable in the five other nerves. On day 72, distal CMAPs, previously undetectable, were measured at 5.3 and 2.6 mV in the median nerves, and 1.1 mV in the left ulnar nerve. CMAP amplitude of the right ulnar nerve was increased by 512%. Partial CBs, defined as > 30% decrease of proximal CMAP amplitude without abnormal temporal dispersion of proximal CMAP (< 30% increased CMAP duration) [8], emerged in intermediate nerve segments of both median and ulnar nerves with decrements of proximal CMAP amplitudes ranging from 30.2 to 73% (Fig. 1). An additional partial CB was detectable in the right median nerve in the Erb’s point-axilla segment (Fig. 1). MCVs were slow. SNAPs with reduced amplitude were recordable in 7/8 nerves. On day 128, distal CMAP amplitudes furtherly increased with however persistently low distal CMAP amplitudes for the common peroneal nerves. Partial CBs improved in the intermediate segments of left median and right ulnar nerves.

**Table 1:** Nerve conduction studies

Nerve	cMAP (mV)	Proximal/Distal CMAP Amplitude (%)	Motor Latencies (ms)	Proximal/Distal CMAP Duration (%)'	CV (m/s)	SNAP (μV)
Right Median (ABP)						
Day 4						
Wrist	NR	NR	NR	NR	NR	NR
Elbow	NR	NR	NR	NR	NR	
Axilla	NR	NR	NR	NR	NR	
Erb’s Point	NR		NR			
Day 72						
Wrist	5,3	69,8	3,79	73,7	31,8	2,1
Elbow	3,7	83,8	11,8	105,3	36,2	
Axilla	3,1	64,5	13,6	125,4	35,2	
Erb’s Point	2		22,4			
Day 128						
Wrist	8,3	59	4	98,4	39,3	3,6
Elbow	4,9	93,9	11	92,3	43,8	
Axilla	4,6	54,3	12,6	120	44	
Erb’s Point	2,5		17			
Day 240						
Wrist	8,5	91,8	3,92	100	43,2	7,5
Elbow	7,8	97,4	10,4	104	50	
Axilla	7,6	72,3	12,2	113,7	36,2	
Erb’s Point	5,5		18			
Left Median (ABP)						
Day 4						
Wrist	NR	NR	NR	NR	NR	NR
Elbow	NR	NR	NR	NR	NR	
Axilla	NR	NR	NR	NR	NR	
Erb’s Point	NR		NR			
Day 72						
Wrist	2,6	47,6	4,54	75,8	29,3	NR
Elbow	1,24	84,7	11,5	101,4	25,6	
Axilla	1,05		15,2			
Day 128						
Wrist	5	62	3,26	91,3	31,2	3,9
Elbow	3,1		11,6NR			
Day 240						
Wrist	6,2	83,8	3,26	92,1	36,6	7,2
Elbow	5,2	100	10,5	106,8	64,3	
Axilla	5,2	92,3	11,9	109,5	41,8	
Erb’s Point	4,8		18			
Right Ulnar (ADM)						
Day 4						
Wrist	0,8	75	4,1	95,3	49	NR
Below Elbow	0,6	100	7,8	108	47	
Above Elbow	0,6	83,3	9,5	95,5	50	
Axilla	0,5		11,3			
Day 72						
Wrist	4,1	27,3	3,74	128	35,6	5,2
Below Elbow	1,12	71,4	10,9	117,8	38,2	
Above Elbow	0,8		13,5			
Day 128						
Wrist	5,7	45,6	2,3	92,2	40,1	5,4
Below Elbow	2,6	80,8	8,54	102,8	37,6	
Above Elbow	2,1		11,2			
Day 240						
Wrist	6,8	86,7	2,03	107,7	43,8	6,7
Below Elbow	5,9	81,3	7,63	107,1	39,3,	
Above Elbow	4,8	79,2	10,3	108,3	38,5	
Axilla	3,8		11,6			
Left Ulnar (ADM)						
Day 4						
Wrist	NR	NR	NR	NR	NR	NR
Below Elbow	NR	NR	NR	NR	NR	
Above Elbow	NR	NR	NR	NR	NR	
AXILLA	NR		NR			
Day 72						
Wrist	1,12	41,9	4,54	98,5	30,9	3,3
Below Elbow	0,47	65,9	11,5	97	32	
Above Elbow	0,31		15,2			
Day 128						
Wrist	3,1	42,2	2,75	83,5	32	3
Below Elbow	1,31	91,6	9,94	100	21,5	
Above Elbow	1,2		11,8			
Day 240						
Wrist	5,1	80,3	2,89	113,3	48,4	5,7
Below Elbow	4,1	80,5	8,06	111,8	34,7	
Above Elbow	3,3	69,7	10,8	107	47,1	
Right Peroneal (EDB)						
Day 4						
Ankle	0,9	22,2	5,9	125,2	56	NR
Below fibular head	0,2	50	11,5	83,5	46	
Above fibular head	0,1		13,5			
Day 72						
Ankle	0,75	49,3	8,13	104,4	25,3	2,1
Below fibular head	0,37	81	20,8	91,5	25	
Above fibular head	0,3		23			
Day 128						
Ankle	0,92	71,7	4,04	97	29,4	2,7
Below fibular head	0,66	87,9	14,6	95,3	36,1	
Above fibular head	0,58		16,4			
Day 240						
Ankle	3,3	78,7	3,9	94,2	33,3	11,6
Below fibular head	2,6	100	13,2	93,9	42,9	
Above fibular head	2,6		15,3			
Left Peroneal (EDB)						
Day 4						
Ankle	NR	NR	NR	NR	NR	NR
Below fibular head	NR	NR	NR	NR	NR	
Above fibular head	NR		NR			
Day 72						
Ankle	NR	NR	NR	NR	NR	2,6
Below fibular head	NR	NR	NR	NR	NR	
Above fibular head	NR		NR			
Day 128						
Ankle	0,24	91,6	6,35	94,6	29,8	4
Below fibular head	0,22	81,8	18,1	90	32,3	
Above fibular head	0,18		19,7			
Day 240						
Ankle	1,77	61	5,34	90	31,1	6,3
Below fibular head	1,08	100	15,3	104,7	34,2	
Above fibular head	1,08		17,2			
RighT sural						
Day 4						
Lateral malleolus						3
Day 72						
Lateral malleolus						3,2
Day 210						
Lateral malleolus						6,6
Left sural						
Day 4						
Lateral malleolus						NR
Day 72						
Lateral malleolus						3,3
Day 240						
Lateral malleolus						9

Control Values: median nerve: distal CMAP amplitude ≥5 mV, DML ≤ 4 ms, MCV ≥ 45 m/s; ulnar nerve: distal CMAP amplitude ≥6 mV, DML ≤ 3,5 ms, MCV ≥ 45 m/s; peroneal nerve: distal CMAP amplitude ≥3 mV, DML ≤ 5,5 ms, MCV ≥ 40 m/s; tibial nerve: distal CMAP amplitude ≥5 mV, DML ≤ 6 ms. Sensory Action Potential Amplitude: ≥ 10 μV for all nerves

*Abbreviations*: *APB* abductor pollicis brevis, *ADM* abductor digiti minimi, *EDB* extensor digitorum brevis, *AH* abductor hallucis, *CMAP* compound muscle action potential, *DML* distal motor latency, *CV* conduction velocity, *SNAP* sensory nerve action potential, *NR* not recordable

The last study on day 240 showed distal CMAP amplitudes in the normal range, except in the left peroneal nerve as well as the resolution of CBs in all nerves with the exception of the emergence of a partial CB in the intermediate segment of the left peroneal nerve, possibly revealed by the increased distal CMAP amplitude due to the resolution of a distal CB. MCV were still slow in some nerve segments. SNAPs amplitudes remained reduced reaching the normal range in only 1/8 nerves.

## Discussion and conclusions

The patient we describe presented with signs suggesting MFS but rapidly developed impaired consciousness and tetraplegia indicating an overlap with BBE and GBS. IgG anti-GQ1b were detected and serology documented a recent *Mycoplasma Pneumoniae* infection. MFS overlapping with GBS or BBE has been reported respectively in 15 and 12% of cases but the triple overlap has rarely been described [6, 7]. Prior *Mycoplasma Pneumoniae* infection has been reported in GBS, MFS and also in BBE [9]. A common denominator to MFS, BBE and GBS is the presence of anti-GQ1b antibody [6].GQ1b gangliosides are highly expressed in the nodal and paranodal regions of the oculomotor nerves, muscle spindle afferents, peripheral nerves and possibly in the brainstem reticular formation [10, 11]. The presence of anti-GQ1b antibodies may explain the complex symptomatology of the patient we report. The overlap among MFS, BBE, and GBS reemphasizes that these disorders are all part of a wide clinical spectrum [12]. The patient presented electrophysiologically with severely reduced or non-recordable CMAPs and SNAPs without demyelinating features indicating, at first test, the electrodiagnosis of acute motor and sensory axonal neuropathy with axonal degeneration and suggesting, in presence of tetraplegia, a poor prognosis. However, distal CMAPs greatly improved in 10 weeks suggesting the resolution of distal CBs whereas CBs in intermediate and proximal nerve segments emerged and unusually persisted for four to 7 months without the development of excessive temporal dispersion of CMAPs, which is considered one of the electrophysiological correlates of de-remyelination [13, 14]. These electrophysiological features and the fact that the resolution of CBs mirrored the clinical improvement suggest that weakness was due to a sustained, antibody-mediated attack at the nodal region inducing a non-demyelinating conduction failure [15–17]. CBs in axonal GBS predominate where the blood-nerve barrier is anatomically deficient, as in the nerve endings, nerve roots, and the common entrapment sites [15, 18]. We hypothesize that, in the patient we report, the improving of distal conduction blocks revealed already existing proximal conduction blocks that persisted for months [8]. Alternatively, the persistence of the autoimmune attack, supported by the high rate of IgG anti GQ1b 5 months after the onset of symptoms, might have caused the appearance of new CBs in more proximal sites.In this last case, other humoral factors present in our patient’s serum probably intervened favoring the disruption of the blood-nerve barrier [19].

Currently, axonal GBS subtypes and MFS are thought to represent acute autoimmune nodopathies with a common pathogenic mechanism characterized by anti-gangliosides mediated dysfunction/disruption of the NAv channel clusters at the Ranvier node and by an electrophysiological continuum from conduction failure, reversible in few weeks, to axonal degeneration [20, 21]. Acute onset and long lasting CBs as in the patient we report, have been described in few patients with IgM antibodies against GM1, GD1a or GD1Q and variably categorized as acute variant of multifocal motor neuropathies or chronic forms of acute motor conduction block neuropathies [22–24]. In neuropathies with anti-ganglioside antibodies it is not explained why most patients have an acute onset with conduction failure that may be promptly reversible or progress to axonal degeneration whereas few others are characterized by persistent CBs. In addition to the duration of the immune attack, intrinsic properties of anti-ganglioside antibodies, such as isotype, affinity and capability to activate complement may be implicated.

Notably, except in the first electrophysiological study, the patient showed slowing of nerve conduction that is commonly assumed to be a characteristic of a de-remyelinating process. However, inactivation of Nav channels by intravenous infusion of lidocaine or tetrodotoxin intoxication reduces conduction velocity, even reaching the “demyelinating” range, possibly by increasing the rise time of the action potential and the internodal conduction time [25, 26]. These findings may well explain the occurrence of slow conduction velocity in an autoimmune neuropathy targeting the excitable axolemma of the node of Ranvier as in the patient we describe [27].

In conclusion, in this patient with an unusual and severe clinical overlap of MFS, BBE, and GBS, serial electrophysiological studies allowed to understand the underlying pathophysiology as attributable to an acute onset, long lasting, nodopathy. Moreover, serial electrophysiology allowed by the second study to formulate a more correct prognosis and guided the treatment as the persistence of CBs encouraged us to carry out a third cycle of IVIg which possibly accelerated the clinical recovery and prevented further axonal degeneration.

## Abbreviations

BBE: Bickerstaff Brainstem Encephalitis
CB: Conduction block
CMAP: Compound muscle action potential
DML: Distal motor latency
ENMG: Electroneuromyography
GBS: Guillain Barré syndrome
IgG: immunoglobulin G
IVIg: Intravenous immunoglobulin
MCR scale: Medical Research Council scale
MCV: Motor conduction velocity
MFS: Miller-Fischer syndrome
RCF: Reversible conduction failure
SNAP: Sensory nerve action potential
